# A Brave New World for an Old World Pest: *Helicoverpa armigera* (Lepidoptera: Noctuidae) in Brazil

**DOI:** 10.1371/journal.pone.0080134

**Published:** 2013-11-18

**Authors:** Wee Tek Tay, Miguel F. Soria, Thomas Walsh, Danielle Thomazoni, Pierre Silvie, Gajanan T. Behere, Craig Anderson, Sharon Downes

**Affiliations:** 1 Biosecurity Flagship, CSIRO Ecosystem Sciences, Canberra, Australian Capital Territory, Australia; 2 Department of Entomology, Instituto Mato-grossense do Algodão – IMAmt (Mato Grosso Cotton Institute), Primavera do Leste, Mato Grosso, Brazil; 3 Sustainable Agricultural Flagship, CSIRO Ecosystem Sciences, Canberra, Australian Capital Territory, Australia; 4 Unité Propre de Recherche Systèmes de culture annuels, CIRAD, Montpellier, France; 5 Unité de Recherche 072, Laboratoire Evolution, Génomes et Spéciation, IRD, Orsay, France; 6 Division of Crop Improvement, Indian Council of Agricultural Research, Research Complex for North Eastern Hill Region, Shilong, Meghalaya, India; 7 Sustainable Agriculture Flagship, CSIRO Ecosystem Sciences, Narrabri, New South Wales, Australia; Bangor University, United Kingdom

## Abstract

The highly polyphagous Old World cotton bollworm *Helicoverpa armigera* is a quarantine agricultural pest for the American continents. Historically *H. armigera* is thought to have colonised the American continents around 1.5 to 2 million years ago, leading to the current *H. zea* populations on the American continents. The relatively recent species divergence history is evident in mating compatibility between *H. zea* and *H. armigera* under laboratory conditions. Despite periodic interceptions of *H. armigera* into North America, this pest species is not believed to have successfully established significant populations on either continent. In this study, we provide molecular evidence via mitochondrial DNA (mtDNA) cytochrome oxidase I (COI) and cytochrome *b* (Cyt *b*) partial gene sequences for the successful recent incursion of *H. armigera* into the New World, with individuals being detected at two sites (Primavera do Leste, Pedra Preta) within the State of Mato Grosso in Brazil. The mtDNA COI and Cyt *b* haplotypes detected in the Brazilian *H. armigera* individuals are common throughout the Old World, thus precluding identification of the founder populations. Combining the two partial mtDNA gene sequences showed that at least two matrilines are present in Brazil, while the inclusion of three nuclear DNA Exon-Primed Intron-Crossing (EPIC) markers identified a further two possible matrilines in our samples. The economic, biosecurity, resistance management, ecological and evolutionary implications of this incursion are discussed in relation to the current agricultural practices in the Americas.

## Introduction

Biological incursions can be natural (e.g., wind-borne fungal spores [1]), intentional (e.g., cane toads (*Bufo marinus*) in Australia [2]; Africanized honeybees in South America [3]) or accidental (e.g., global invasion of the red imported fire ant (*Solenopsis invicta*) [4]; brown marmorated stink bug (*Halyomorpha halys*) and kudzu bug (*Megacopta cribraria*) in the US [5]–[10]; boll weevil (*Anthonomus grandis*) in Brazil [11]–[13]), with accidental introductions usually associated with human activities (e.g., global trade [14]). Invasive species are not necessarily readily recognised especially when introduced into an environment where closely related species exist, and may displace other congeners [15], [16]. Rapid confirmation of such incursions is imperative and can be greatly assisted by characterisation of known genomic regions.

Global trade is rapidly diminishing the effective distance between countries and border biosecurity via quarantine inspections represents the last line of defence. Once breached, the establishment of invasive species can lead to rapid and significant negative economic and environmental impacts. The Old World cotton bollworm *Helicoverpa armigera* (Lepidoptera: Noctuidae) is the most significant and impactful pest of agriculture in Asia, Europe, Africa and Australasia, causing damage to crops estimated at greater than US$2 billion annually, excluding socio-economic and environmental costs associated with its control. Although restricted to the old world, *H. armigera* has long been recognised as a serious biosecurity threat to the Americas, where it has the potential to establish across up to 49% of the North American continent with far greater anticipated potential economic loss to corn and cotton than that of *H. zea*, [17], [18]. *H. armigera* is regularly intercepted at U.S. ports-of-entry (e.g., 20 times in 2003) on a variety of cut flowers and herbs originating from countries in the Old World (e.g., Bosnia, Israel, Japan, The Netherlands, New Zealand, Zimbabwe) [18], and with 4,431 interceptions (annual rate of *ca*. 280±12 standard error) since 1985 for *H. armigera* or “*Helicoverpa* sp.” at the US “first points of entry” [17]. Given the global nature of many prime host crops, the establishment of *H. armigera* in the Americas would pose a severe and continuing threat to many crops in the New World. In Brazil *H. armigera* has been recognized as a quarantine pest since 1999, and received the A1 quarantine pest status in 2008 [19]–[21].

In addition to feeding on an extensive range of hosts (>180 plant hosts from >45 families), including essential global food and fibre crops, *H. armigera* has repeatedly developed rapid resistance to insecticides (e.g., [22]–[24]). *H. zea* is similarly polyphagous but demonstrates lesser propensity than *H. armigera* for damage and resistance development. *H. zea* is thought to be derived from *H. armigera* founders approximately 1.5 – 2 million years ago and the difference in economic damage and resistance development may be the consequences of a genetic bottleneck [25].

In Australia there is considerable experience in managing the threat to agriculture posed by *H. armigera* but despite a pre-emptive Insecticide Resistance Management (IRM) strategy, field populations of *H. armigera* developed levels of resistance that reduced the effectiveness of products from all of the commonly used groups of chemistries deployed for their management [22]. *H. armigera* is the primary target of genetically modified cotton, containing insecticidal proteins from *Bacillus thuringiensis* (Bt) cotton which was introduced to Australia in the mid-1990's largely to overcome insecticide resistance issues. However, from the outset high baseline levels of resistance were detected in this pest to one of the Bt toxins in current varieties and resistance risk remains a critical concern [26]. Early detection of field-evolved resistance to Bt cotton was recently reported for *H. armigera* in China [27].

Since the expansion of agriculture in the late 1970's in Brazil, a complex of insects and mites have established as pests that can contribute to significant yield losses. Noctuids (Lepidoptera: Noctuidae) are amongst the most destructive pest family. Specifically, intensive cultivation of soybeans, corn and cotton, which for the past 20 years has characterized the Cerrado region (Brazilian Savannah), is affected by an inter-crop pest complex composed of *Chrysodeixis includens, Spodoptera frugiperda, S. cosmioides, S. eridania*, *Heliothis virescens* and *Helicoverpa* spp. [28]. Control of this pest complex is difficult due the broad range of hosts in the landscape including vast areas of non-monitored uncultivated plants which serve as refuges from chemical control, and sources for recolonisation.

In the 2011/2012 and 2012/2013 growing seasons, high infestations of *Helicoverpa* species larvae were detected in different regions of Brazil attacking row and cover crops, resulting in significant economic losses. Initially the species was presumed to be *H. zea*, which is often found in maize and tomato crops in Brazil [29]. However, large numbers of row crops were attacked consecutively in the same agricultural landscape at unusually high infestations, and growers reported a reduced efficacy of different methods of control for the pest. Consequently, investigations took place to determine if the species was indeed *H. zea* as presumed or perhaps another species such as *H. armigera*.

Recent reports suggest that *H. armigera* has been identified in Brazil with notifications about its incidence based on morphological assessments of *Helicoverpa* spp. specimens [30], [31]. Species identification based on morphological characters between *H. armigera* and *H. zea* is difficult. Identification via male genitalia morphology has been used extensively however Pogue [18] reported overlapping ranges in male vesica between *H. armigera* and *H. zea*. Well-characterised molecular markers [32], [33] can be used to confirm if this Old World pest has successfully established in the New World. This study reports for the first time at a molecular level that *H. armigera* is in Brazil and that there are at least four maternal lineages present. We discuss the potential implications of incursions of *H. armigera* in Brazil and throughout the New World.

## Results

We analysed 14 lepidopteran samples collected in 2013 from Mato Grosso in Brazil using two standard mtDNA markers that have been shown to effectively differentiate the four major *Helicoverpa* pest species including *H. armigera* and *H. zea* [32]. We successfully obtained PCR amplicons for partial mtDNA COI and Cyt *b* regions for 11 of the 14 specimens. Of the 11 COI amplicons sequenced, six samples (four adult moths from Southern Mato Grosso, and two larvae from Central-Eastern Mato Grosso) were identified as *H. armigera* with mtDNA COI sequences (KF150288 to KF150293) that matched the COI-Harm01 haplotype (EF116226.1, e-value: 0.0, Identity: 510/510, 100%) of Behere et al. [25], thereby confirming the presence of this species in Brazil. The COI-Harm01 haplotype is the most common global *H. armigera* COI haplotype, present in all sampled regions in India (10 sites), Africa (2 sites), Australia (3 sites), China (1 site), and Pakistan (1 site) [25]. Two specimens were identified as *Spodoptera frugiperda* (KF150294, KF150295) matching HQ177352.1 for this species (e-value: 0.0; 99% identity (463/467)), and two were identified as *S. eridania* (KF150296, KF150297) that matched three existing GenBank entries for this species (HQ177329.1, HQ177322.1, and HQ177321.1; e-value: 0.0, 99% identity (465/467)); both species are established pests in the Americas. One sample was an unknown lepidopteran species with COI partial sequence (432bp, KF150298) that best matched two GenBank entries (AJ420367.1 and AJ420368.1) for *Diachrysia tutti* (Noctuidae: Plusiinae) (e-value: 3e-176, Identities: 398/432, 92%). The remaining three of the 14 lepidopteran samples failed to generate PCR products for both COI and Cyt *b*, possibly due to poor DNA quality or due to these samples being too divergent from the mtDNA COI and Cyt *b* markers used in this study.

**Figure 1:**
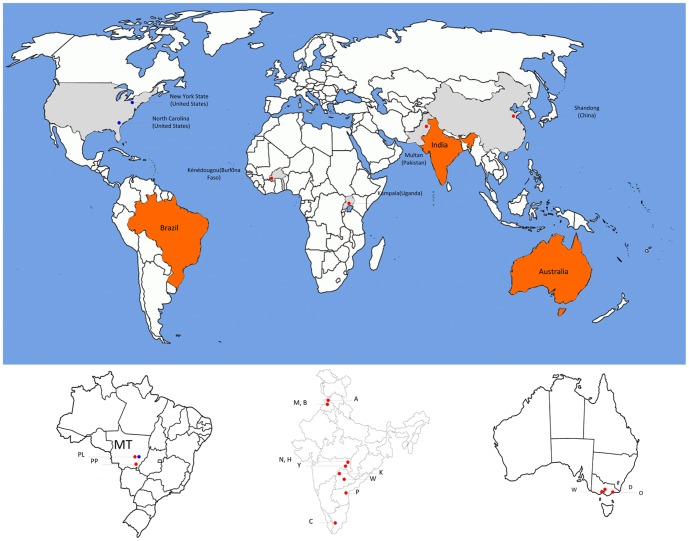
A map of sampling sites and countries from which *Helicoverpa armigera* and *H*. *zea* were obtained for this study. Three populations of *H*. *zea* (blue filled circles) were from the Mato Gosso (MT) state of Brazil at Primavera do Leste (PL), and from New York State and North Carolina (United States). Sites where *H*. *armigera* were sampled are represented by red filled circles, and included the African continent (Burkina Faso and Uganda), India, China (Shandong Province) and Australia. Detailed sites within India were Abohar (A), Mansa (M), Bhatinda (B), Nagpur (N), Hingoli (H), Yavatmal (Y), Karimnagar (K), Warangal (W), Prakasam (P), and Coimbatore (C). Within Australia the Warribee (W), Dalmore (D) and Orbost (O) populations were from the state of Victoria. Individuals of *H*. *armigera* detected from Brazil in this study were from the MT state at Primavera do Leste (PL) and at Pedra Preta (PP).

**Figure 2:**
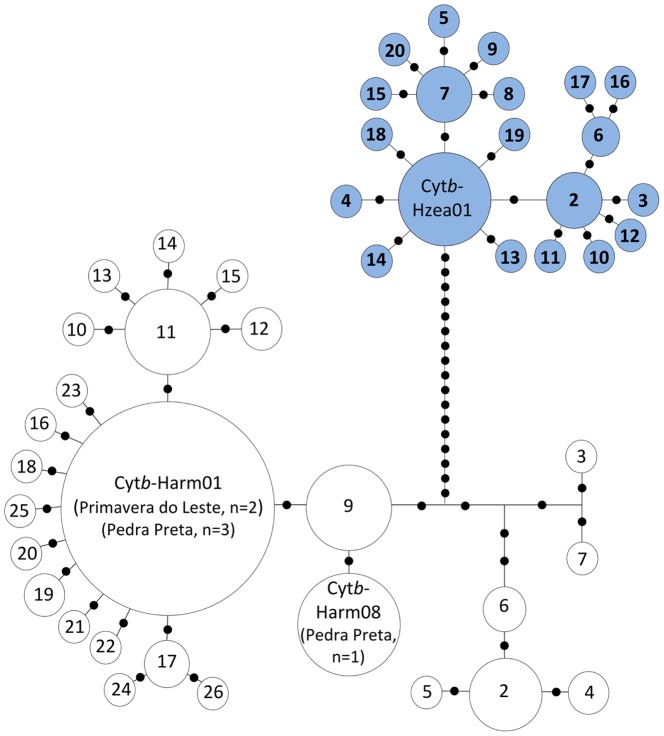
Mitochondrial DNA Cyt *b* haplotype network *of Helicoverpa armigera* (white circles) and *H. zea* (blue circles) based on 434bp of partial Cyt *b* gene. A total of 26 *H. armigera* haplotypes were identified from 255 individuals sampled from India (n = 90), Pakistan (n = 10), China (n = 34), Burkina Faso (35), Uganda (24), Australia (56), and Brazil (6), and a total of 20 *H. zea* Cyt *b* haplotypes from 64 individuals were sampled from Brazil (30), and the US (North Carolina (14), New York (20). *H. armigera* and *H. zea* cyt *b* haplotypes are as provided in Table S1. Numbers of *H*. armigera individuals within each cyt*b*-Harm haplotype are: 1 (n = 156), 2 (n = 10), 3(n = 1), 4 (n = 2), 5 (n = 1), 6 (n = 3), 7 (n = 1), 8 (n = 23), 9 (n = 17), 10 (n = 1), 11 (n = 20), 12 (n = 2), 13–16 (n = 1 each), 17 (n = 4), 18 (n = 1), 19 (n = 2), 20–26 (n = 1 each). Numbers of nucleotide substitutions differentiating between haplotypes are indicated by black circles (e.g., total of 18 nucleotide substitutions separate the *H. armigera* Cyt*b*-Harm09 haplotype from the *H. zea* Cyt*b*-Hzea01 haplotype). The Brazilian *H. armigera* possessed the Cyt*b*-Harm01 (n = 2, from Primavera do Leste; n = 3 from Pedra Preta) and Cyt*b*-Harm08 (n = 1, from Pedra Preta).

The partial Cyt *b* region of the six *H. armigera* individuals indicated the presence of two mtDNA Cyt *b* haplotypes. Five individuals were Cyt*b*-Harm01 (KF150299 to KF150303) and 1 adult individual from the southern region was Cyt*b*-Harm08 (KF150304), providing evidence that the *H. armigera* populations in Brazil consisted of multiple maternal lineages. The Brazilian *H. armigera* Cyt*b*-Harm01 haplotype matched the reported sequence EF410020.1 of Behere et al. [33], which was the most prevalent haplotype across the 17 global sites ([25]; Table S1), while the Brazilian *H. armigera* Cyt*b*-Harm08 matched the Behere et al. [33] global Cyt*b*-Harm08 haplotype (EF410027.1) (e-value: 0.0; Identity: 388/388, 100%) that was only detected in Bhatinda and Warangal (India), Kenedougou (Burkina Faso), Kampala (Uganda), Orbost (Australia), and Shandong (China) (Table S1). A map of countries and locations of samples reported in this study is presented in Fig. 1. A Cyt *b* haplotype network linking *H. zea* populations from the US (North Carolina and New York) and Brazil (Mato Grosso, Primavera do Leste) with *H. armigera* global populations is also presented (Figure 2). The Cyt *b* haplotype network showed two nucleotide substitutions separating the Cyt*b*-Harm01 and Cyt*b*-Harm08 haplotypes where the current Brazilian *H. armigera* Cyt *b* haplotypes were detected. A total of 18 nucleotide substitutions separated the *H. armigera* Cyt*b*-Harm09 haplotype and the *H. zea* Cyt*b*-Hzea01 haplotype. The Cyt *b* haplotype network agreed with the mtDNA COI haplotype network of Behere et al. (Figure 1 in [25]) which suggested an African origin of present day *H. zea*, but lacked concordance at a finer spatial scale (i.e., Burkina Faso based on mtDNA COI [25], Uganda based on mtDNA Cyt *b* (Table S1)), although the Cyt*b*-Harm09 haplotype was also detected in Pakistan, India and China (Table S1).

Genotyping using exon-primed intron-crossing (EPIC) PCR markers RpL11, RpS6 and DDC [32], [34] across 18–20 *H. zea* individuals collected in 2006 from Central-Eastern region of Mato Grosso, Brazil [25] indicated homozygous alleles for RpL11 (207 bp/207 bp; n = 20) and RpS6 (270 bp/270 bp; n = 18), while 4 alleles were detected using DDC (206–209 bp; n = 19) [32]. Genotyping of the six Brazilian *H. armigera* individuals indicated that in addition to the individual matching the Cyt*b*-Harm08 which confirmed a second matriline, genotypes of the remaining five individuals that shared the mtDNA COI-Harm01 haplotype could be further explained by there being three different matrilines, assuming single mating in both male and female *H. armigera* (Table S2).

## Discussion

This study confirms via molecular characterisation of mtDNA COI and Cyt *b* genes, that *H. armigera* has successfully established at least two populations in the New World. Previously, thirty *Helicoverpa* larvae collected from maize in 2006 from the Centre-East region of Mato Grosso (Primavera do Leste) were identified as *H. zea* [25], while six of the 11 lepidopteran individuals from the Centre-East and Southern regions of the same State analysed herein were *H. armigera*. Both *H. zea* and *H. armigera* are highly polyphagous and readily attack both maize and cotton [35], [36]. Shifts in patterns of host use, levels of infestation, and efficacy of control methods against *Helicoverpa* spp., suggest that *H. armigera* proliferated sometime in the last two years. In particular, in 2012/13 farmers and consultants reported greater than normal injury and damage on cotton and soybeans in the extreme Western region of Bahia state (BA) and other regions of Northern states. In Mato Grosso, the occurrence of *Helicoverpa* spp. is relatively low compared with BA and it occurs simultaneously with *Heliothis virescens* on soybeans and/or cotton. Approximately 30% of *H. virescens* and *Helicoverpa* spp. moths captured in the field during an 8-week period early in the growing season of 2012/2013 were *Helicoverpa* in the Centre, Centre-East and South of MT, while prevalence and continuous trapping of *Helicoverpa* species moths at the end of the growing season indicated moth populations would likely increase in the next cropping season (M. F. Soria unpublished data).

The detection of *H. armigera* in South America represents the second occasion that this species has occupied the New World. The first entrance was presumably the result of natural spread possibly of African origins [25]. While the most recent invasion confirmed here is likely the result of human activity, there is nevertheless insufficient data to rule out chance natural spread events, since under favourable weather patterns *H. armigera* have been known to migrate up to 2,000 km [37], [38]. Understanding the pathways to incursion by the Old World cotton bollworm is important and may assist in preventing potential incursions by other species such as the Solanaceae specialist *Helicoverpa assulta* from the Old World.

Our molecular analyses based on mtDNA markers suggest that the *H. armigera* populations from Mato Grasso, Brazil, arose from at least two maternal lineages, representing either multiple recent incursions, or a single incursion from mixed populations of the Old World bollworm. EPIC marker analyses of these six individuals confirmed that in addition to a separate maternal lineage as indicated by the mtDNA Cyt*b*-Harm08 haplotype, the remaining five individuals with the most prevalent mtDNA COI-Harm01 haplotype could be further explained by three separate maternal lineages (Table S2). This enables a conservative estimate of at least 4 separate matrilines in our six Brazilian *H. armigera* samples. Our estimates of matrilines in the limited Brazilian *H. armigera* samples assumed single mating and no hybridisation and/or introgression. The availability of a global mtDNA COI and Cyt *b* haplotype database ([25], [33]; Table S1) and extensive sampling and EPIC population genetic study of *H. armigera* from the Old World [34] enable us to explore the possible origins from which the founding individuals originated from. However, we have been unable to define the precise origins of the Brazilian *H. armigera* because they possessed mtDNA COI and Cyt *b* haplotypes that were prevalent in all regions of the Old World.

The implications of a recent invasion of *H. armigera* for Brazilian agriculture are potentially serious, mainly due to its history of rapidly evolving resistance to insecticides [22]–[24], and the year round presence of potential cultivated hosts. Monitoring for pest resistance to insecticides and Bt varieties by researchers independent of technology providers has only recently begun in Brazil. There is no formal co-ordinated strategy for managing resistance to insecticide sprays. Since many populations of *H. armigera* around the world developed resistance to conventional pesticides (see above), it is possible that the founders of the invasive populations include individuals with genetic make-up that predisposes them to insecticide adaptation. There would be strong selection on these individuals to enable them to rapidly exploit agricultural systems where the endemic pests remain susceptible to conventional pesticides.

In addition, the arrival of *H. armigera* in Brazil poses a risk to the extensive Bt agriculture in South America. In many areas in Brazil, Bt crops expressing events from the same toxin class (Cry1) are grown in succession and/or at the same time in various proportions across as much as 30, 15 and 1 million hectares for soybeans, maize, cotton respectively [39], [40], resulting in enormous selection pressure. Currently these crops are grown without the mandated resistance management practices that are adopted elsewhere, including for example, the use of non-Bt refuge crops, and a mechanism to prevent carryover of potentially selected individuals from one crop to the next (e.g., via pupae destruction or use of trap crops; see for example [41]).

A further complication is that, despite the duration of speciation between *H. armigera* and *H. zea*, mating between these two species under laboratory conditions is possible and can generate fertile offspring [42]–[44]. The close evolutionary relationship between these two species is further supported by *H. armigera* males being attracted to the sex pheromones used in traps for field capturing of male *H. zea* [45], and that the same pheromone compounds have been found in the two species [18]. However, hybridisation in the laboratory does not automatically mean that even if given the opportunity to co-exist in the field that hybrid offspring will result. It is also possible that re-introduction of the original progenitor species, *H. armigera*, into the New World will create an event that is equivalent to ‘isolate breaking’, whereby the resultant *zea*-*armigera* hybridisation may lead to heterosis. Genetic fitness in these hybrid offspring may present new challenges for IRM strategies.

Venette et al. [17] assessed movements of commodities and passengers into the US through international airports in various States as likely incursion routes of *H. armigera*. Given detection in South America and an essentially continuous landscape of suitable host crops and climatic condition through Central America into the US, natural dispersion of this serious agricultural pest should be considered, with likely entry being via Mexico and/or through the Caribbean Islands into South-Eastern US. Failure to develop effective control measures for potential arrival of *H. armigera* into the US may have serious economic consequences, adding to existing prohibitively high costs in mitigating introduced pests such as the Africanized honeybee and *S. invicta* that similarly entered the US from South America [46], [47]. An invasion of *H. armigera* would be especially serious if it entered the US from South America with high levels of resistance to insecticides and/or Bt toxins.

The incursion of *H. armigera* into Brazil is unique in that it could have profound biological consequences to the species status and population genetics of *H. zea* and related New World *Helicoverpa* species, the plant-insect interactions and ecology, evolution of insecticide resistance, hybridisation, introgression and heterosis, as well as significant social-economic impact on developing effective integrated pest management (IPM) and IRM strategies in the New World. Addressing these issues will be greatly benefited by knowledge of the *H. armigera* and *H. zea* genomes, which are currently being prepared by the *Helicoverpa armigera* genome consortium led by the CSIRO [48]. Knowing how *H. armigera* re-entered the Americas would assist with global biosecurity preparedness that may minimise opportunities for other pest species to attain global significance.

## Materials and Methods

### Lepidopteran sample collection

Permit access to collect material used in our research at various crop sites was granted by respective growers. Suspect lepidopteran samples were collected from Centre-East region – Primavera do Leste (15°27′57″S 54°17′06″W and 15°32′02″S 54°11′47″W) (1 live adult and 2 live (3^rd^ and 5^th^ instar) caterpillars, collected on 27^th^ March, 2013) and South region – Pedra Preta (16°50′35″S 54°00′23″W) (20^th^ March, 2013: 3 dead adults; 27^th^ March, 2013: 1 dead and 7 live adults) of Mato Grosso (MT) State, Brazil. Live/dead adult moths were collected via light traps set in cotton fields with maize as an adjacent neighbouring crop where soybeans were grown before maize and cotton. The samples were placed in >95% ethanol in individual 1.5mL Eppendorf tubes. Larvae first identified as cotton budworms/bollworms were collected from a Bt cotton variety cultivated inside the experimental station of Instituto Mato-Grossense do Algodão (Mato Grosso Cotton Institute) - IMAmt - and placed in Eppendorf tubes as for the moths. All suspect samples were analysed with no exclusion of any specimen even if believed to be *H*. *zea*.

### Molecular characterisation of partial mitochondrial DNA genes

Molecular characterisation took place at the CSIRO Ecosystem Sciences Black Mountain Laboratories in Australia. Total genomic DNA (gDNA) was extracted for all adult moths and larvae using a Qiagen Blood and Tissue DNA extraction kit. A tissue fragment of each ethanol preserved specimen was cut off with sterilised scissors. An extraction blank was simultaneously processed to ensure that there was no external source of *H. armigera* DNA. To confirm species status, partial mitochondrial DNA (mtDNA) cytochrome oxidase I (COI) and cytochrome *b* (Cyt *b*) gene fragments were amplified from every individual using primers COI-F02/R02 and Cytb-F02/R02 prior to sequencing and assembled as previously described [33]. Sequenced PCR fragments were identified by Blastn and aligned to known mitochondrial DNA sequences. The global *H. armigera* Cyt *b* haplotypes and three populations of *H. zea* Cyt *b* haplotypes were previously reported [33] and their distribution patterns are here presented (Table S1, Fig. 2). A haplotype network for the global mtDNA COI haplotypes has also been reported [25]. The mtDNA Cyt *b* haplotype network was constructed manually and verified using TCS version 1.21 [49].

### Exon-Primed Intron-Crossing (EPIC) PCR marker analyses

We applied the EPIC markers RpL11, RpS6 and DDC as reported in Tay et al. [32] and Behere et al. [34] following protocols described therein. We also included positive controls (one Mato Grasso *H. zea* (individual 13) and one *H. armigera* (CSIRO Ecosystem Sciences general laboratory (GR) rearing strain) in all amplifications as well as negative controls to detect potential cross-contamination. PCR amplicons were first observed under UV-illuminator followed by dilution (1∶100) in sterile water prior to being genotyped by a commercial company (1^st^ Base; <www.base-asia.com>). The fragment patterns were interpreted using Geneious Pro 5.6.5 <www.geneious.com>).

## Supporting Information

Table S1 Global *Helicoverpa armigera* and *H. zea* Cyt *b* haplotype distribution patterns including the Brazilian *H. armigera* Cyt*b*-Harm01 and Cyt*b*-Harm08 haplotypes, and relevant GenBank Accession numbers.
(DOCX)

Table S2 Genotypes of six *Helicoverpa armigera* from Brazil using RpL11, DDC and RpS6 exon-primed intron-crossing (EPIC) PCR markers of Tay et al. [32], with possible numbers of matrilines contributing to the Brazilian *H*. *armigera* being indicated.
(DOCX)
