# Supplementary material for: A Brave New World for an Old World Pest: Helicoverpa armigera (Lepidoptera: Noctuidae) in Brazil
Source: PLoS One. 2013 Nov 18;8(11):e80134. doi: 10.1371/journal.pone.0080134 (PMC3832445; doi:10.1371/journal.pone.0080134)
Supplement: Table S1 — Global Helicoverpa armigera and H. zea Cyt b haplotype distribution patterns including the Brazilian H. armigera Cytb-Harm01 and Cytb-Harm08 haplotypes, and relevant GenBank Accession numbers. (DOCX) [file pone.0080134.s001.docx]

**Supplementary Tables**

**Table S1:** Global *Helicoverpa armigera* and *H. zea* Cyt *b* haplotype distribution patterns including the Brazilian *H. armigera* Cyt*b*-Harm01 and Cyt*b*-Harm08 haplotypes, and relevant GenBank Accession numbers. Brazilian *H. armigera* samples were collected from the Southern region of Mato Grosso State (Pedra Preta) on 20^th^ March (2 adults) and 27^th^ March (2 adults), 2013, from light traps, and on 27^th^ March, 2013, on Bt cotton plants of an experimental field (2 larvae, from the Centre-East region of Mato Grosso State, Primavera do Leste). Host plants and collection dates for all remaining *H. armigera* and *H. zea* samples were as reported in Behere et al. [25]. Numbers of individuals sequenced from each locality are indicated in parentheses.

| **Countries** | **Locations** | **Cyt*b* Haplotypes** | **GenBank Accession numbers** |
| --- | --- | --- | --- |
| ***Helicoverpa armigera*** | | | |
| India | Mansa (5) | Cyt*b*-Harm01, 10, 11 | EF410020.1, EF410029.1, EF410030.1 |
|  | Bhatinda (5) | Cyt*b*-Harm01, 08, 09, 11 | EF410020.1, EF410027.1, EF410028.1, EF410030.1 |
|  | Abohar (5) | Cyt*b*-Harm01 | EF410020.1 |
|  | Yavatmal (10) | Cyt*b*-Harm01, 02, 03, 11 | EF410020.1, EF410021.1, EF410022.1, EF410030.1 |
|  | Hingoli (5) | Cyt*b*-Harm01, 09 | EF410020.1, EF410028.1 |
|  | Nagpur (11) | Cyt*b*-Harm01, 02, 09, 11 | EF410020.1, EF410021.1, EF410028.1, EF410030.1 |
|  | Prakasam (6) | Cyt*b*-Harm01, 09 | EF410020.1, EF410028.1 |
|  | Coimbatore (22) | Cyt*b*-Harm01, 02, 11, 12, 25 | EF410020.1, EF410021.1, EF410030.1, EF410031.1, EF410044.1 |
|  | Karimnagar (10) | Cyt*b*-Harm01 | EF410020.1 |
|  | Warangal (11) | Cyt*b*-Harm01, 08 | EF410020.1, EF410027.1 |
| Burkina Faso | Kenedougou (35) | Cyt*b*-Harm01, 02, 08, 11, 23 | EF410020.1, EF410021.1, EF410027.1, EF410030.1, EF410042.1 |
| Uganda | Kampala (24) | Cyt*b*-Harm01, 02, 04, 08, 09, 15 | EF410020.1, EF410021.1, EF410023.1, EF410027.1, EF410028.1, EF410034.1 |
| Australia | Orbost (24) | Cyt*b*-Harm01, 05, 06, 08, 11, 17, 18 | EF410020.1, EF410024.1, EF410025.1, EF410027.1, EF410030.1, EF410036.1, EF410037.1 |
|  | Dalmore (22) | Cyt*b*-Harm01, 04, 06, 11, 13, 17, 24 | EF410020.1, EF410023.1, EF410025.1, EF410030.1, EF410032.1, EF410036.1, EF410043.1 |
|  | Werribee (10) | Cyt*b*-Harm01, 11, 26 | EF410020.1, EF410030.1, EF410045.1 |
| China | Shandong (34) | Cyt*b*-Harm01, 06, 07, 08, 09, 11, 14, 16, 19, 20, 21, 22 | EF410020.1, EF410025.1, EF410026.1, EF410027.1, EF410028.1, EF410030.1, EF410033.1, EF410035.1, EF410038.1, EF410039.1, EF410040.1, EF410041.1 |
| Pakistan | Multan (10) | Cyt*b*-Harm01, 09 | EF410020.1, EF410028.1 |
| Brazil | Mato Grosso, Primavera do Leste (2) | Cyt*b*-Harm01 | KF150302, KF150303 |
|  | Mato Grosso, Pedra Preta (4) | Cyt*b*-Harm01, 08 | KF150299, KF150300, KF150301, KF150304 |
| ***Helicoverpa zea*** | | | |
| USA | North Carolina (14) | Cyt*b*-Hzea01, 02 | EF410059.1, EF410060.1 |
|  | New York (20) | Cyt*b*-Hzea01, 02, 03, 04, 05, 06, 07, 08, 09, 10, 11, 12, 13, 14 | EF410059.1, EF410060.1, EF410061.1, EF410062.1, EF410063.1, EF410064.1, EF410065.1, EF410066.1, EF410067.1, EF410068.1, EF410069.1, EF410070.1, EF410071.1, EF410072.1 |
| Brazil | Mato Grosso, Primavera do Leste (30) | Cyt*b*-Hzea01, 07, 09, 15, 16, 17, 18, 19, 20 | EF410059.1, EF410065.1, EF410067.1, EF410073.1, EF410074.1, EF410075.1, EF410076.1, EF410077.1, EF410078.1 |
