# Supplementary material for: A Brave New World for an Old World Pest: Helicoverpa armigera (Lepidoptera: Noctuidae) in Brazil
Source: PLoS One. 2013 Nov 18;8(11):e80134. doi: 10.1371/journal.pone.0080134 (PMC3832445; doi:10.1371/journal.pone.0080134)
Supplement: Table S2 — Genotypes of six Helicoverpa armigera from Brazil using RpL11, DDC and RpS6 exon-primed intron-crossing (EPIC) PCR markers of Tay et al. [32], with possible numbers of matrilines contributing to the Brazilian H. armigera being indicated. (DOCX) [file pone.0080134.s002.docx]

**Table S2:** Genotypes of six *Helicoverpa armigera* from Brazil, one *H. zea* sampled in 2006 from Mato Grasso (MT), and an Old World *H. armigera* (CSIRO Ecosystem Science laboratory general rearing (GR) strain) using RpL11, DDC and RpS6 exon-primed intron-crossing (EPIC) PCR markers of Tay et al. [32]. Allele sizes are in base pair (bp). Alleles that failed to amplify are indicated by 'x’, and maybe due to poor gDNA quality and/or null alleles [32, 34], the result of incompatibility between markers sequences at primer annealing sites. Failed amplifications have not affected the conservative estimates of number of female founders. Mitochondrial DNA (mtDNA) COI and Cyt *b* haplotypes of test subjects are also provided for the Brazilian *H. armigera*) and for *H. zea*-individual 13 (COI-Hzea06 (EF116264.1) [25], Cyt*b-*Hzea01 (EF410059.1)) from MT, but not determined (N.D.) for the GR *H. armigera* control. Possible numbers of matrilines contributing to the Brazilian *H*. *armigera* are indicated.

| **Sample** | **COI** | **Cyt *b*** | **RpL11**  **(bp)** | **DDC**  **(bp)** | **RpS6**  **(bp)** | **Possible Matrilines** |
| --- | --- | --- | --- | --- | --- | --- |
| Brazil-04 | Harm01 | Harm01 | 297/297 | 208/218 | 266/268 | 1 |
| Brazil-07 | Harm01 | Harm01 | 297/297 | 208/208 | 269/271 | 1 |
| Brazil-10 | Harm01 | Harm01 | 297/297 | 206/206 | 270/275 | 2 |
| Brazil-11 | Harm01 | Harm08 | 297/297 | 211/213 | 265/271 | 3 |
| Brazil-13 | Harm01 | Harm01 | 297/297 | x/x | x/x | N.D. |
| Brazil-14 | Harm01 | Harm01 | x/x | 204/221 | 247/247 | 4 |
| *H. zea*-13 (MT) | Hzea06 | Hzea01 | 297/297 | 206/206 | 270/270 | Control |
| *H. armigera* (GR) | N.D. | N.D. | x/x | 204/206 | 264/270 | Control |

**Note:** Brazil-04 and -07 are consistent with being offspring from mating between adult-1 of RpL11 (297/297), DDC (208/208), RpS6 (any two alleles of 266/268/269/271), and adult-2 of RpL11 (297/297), DDC (208/218), RpS6 (the remaining two alleles). Brazil-10 shared an allele for each of the three loci with the control MT *H. zea*, and has unique alleles at the RpS6 locus. Brazil-11 is the offspring from a third female as indicated by the Cyt*b*-Harm08 unique haplotype. Brazil-14 has unique alleles for the DDC locus that are not present in matrilines 1, 2 or 3.
